# Corneal perforation in ocular cicatricial pemphigoid: A CARE-compliant case report

**DOI:** 10.1097/MD.0000000000028266

**Published:** 2021-12-23

**Authors:** Suan Hwang, Shu-Chun Kuo

**Affiliations:** a Department of Ophthalmology, Chi Mei Medical Center, Tainan, Taiwan; b Department of Optometry, Chung Hwa University of Medical Technology, Tainan, Taiwan.

**Keywords:** amniotic membrane, amniotic membrane transplantation, mucous membrane pemphigoid, ocular cicatricial pemphigoid

## Abstract

**Rationale:**

Ocular cicatricial pemphigoid (OCP) is a potentially blinding, rare systemic autoimmune disease. The definite etiology of OCP remains under debate, early diagnosis is important to prevent rapid deterioration. The majority of the discussion has been focused on its medical therapeutic strategy, while little effort has been made to study the role of amniotic membrane transplantation (AMT). We describe the first case of OCP with minimal immunosuppressant and initial ocular surface reconstruction procedure using double layer AMT.

**Patient concerns:**

A 66-year-old female patient presented to our outpatient department with right eye pain for several days.

**Diagnosis:**

Slit lamp examination revealed the right eye cornea perforation with iris incarceration and total collapse of anterior chamber. Symblepharon formation and severe fornix shortening was also noted. While bulbi phthisis with ankyloblepharon and ocular surface keratinization was observed in the left eye. The final diagnosis was right eye stage III ocular cicatricial pemphigoid complicated with corneal perforation and iris prolapsed.

**Interventions:**

The patient underwent ocular surface reconstruction with the aid of amniotic membrane. The first layer of the amniotic membrane was attached with tissue adhesive and fibrin glue while the second layer amniotic membrane came with a conformer ring which supported the fornix space that was recreated. Postoperative care included topical medications for inflammation alleviation. Systemic immunosuppressive agents were not administered except for oral prednisolone.

**Outcomes:**

No recurrence of symblepharon was noted during the one year follow-up.

**Lessons:**

We aim at highlighting the possible important role of AMT in advance OCP. Further investigation is still needed for providing evidence to incorporate the procedure into treatment protocol.

## 1 Introduction

Ocular cicatricial pemphigoid (OCP) is a systemic autoimmune disease, which rarely affects the eyes. The disease has a reported incidence of 0.8 per million population and an average age at disease onset of 65 years.^[1,2]^ OCP is usually characterized by corneal subepithelial blistering, conjunctival inflammation and eventually scarring.^[3]^ Early diagnosis and treatment play important roles in preventing rapid deterioration, although lid abnormalities such as trichiasis, entropion, and lagophthalmos due to the exposure and abrasion of the corneal surface may lead to misdiagnosis. Wide-ranging multimodal therapeutic strategy, including systemic immunosuppressive therapy, is crucial in halting inflammation in patients with OCP, but the side effects of the immunosuppressive agents are significant, especially in the elderly.^[4]^ Herein, we present a rare case of a 66-year-old female with corneal perforation due to severe OCP and aim to describe the important role of self-retained cryopreserved amniotic membrane in the reconstruction and maintenance of the ocular surface.

## 2 Case report

A 66-year-old female patient presented to our ophthalmology outpatient department with right eye pain for several days. She denied history of facial trauma or the presence of a foreign body in the right eye. The patient did receive left eye therapeutic penetrating keratoplasty ten years ago with graft failure and eventually became bulbi phthisis. Additionally, due to a massive subfoveal hemorrhage related to age-related macular degeneration, right eye received several intravitreal injections of Bevacizumab and Aflibercept before this presentation. Optical coherence tomography at that time showed subretinal fibrosis at the macular of her right eye and the best corrected visual acuity was hand motion. Except for these ocular related medical history, no other significant medical, social, or family history was noted.

**Figure 1:**
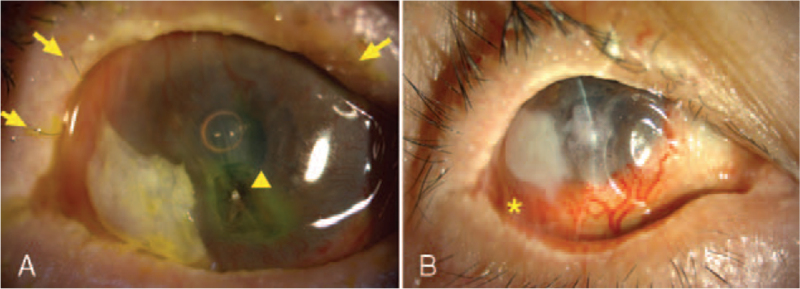
Slit lamp photograph of the right eye. (A) A bandage contact lens was applied. Central melting of the corneal with iris incarcerated (arrowhead), corneal neovascularization, and trichiasis (arrow) could be seen. (B) Symblepharon at the inferior fornix limited the ocular movement. (asterisk).

According to the patient, she could only recall an event of sudden severe ocular pain in the right eye three years ago, which was three months after to her cataract surgery. A large central corneal epithelial bulla rupture was found at that time. This time, she experienced sudden sensations of pain which similar to the previous incident. Thus, she visited our department for further evaluation. The right eye was the only functional eye with positive light perception. Slit-lamp examination showed a paracentral corneal melting, perforation with incarcerated iris, and anterior chamber collapsed. Moreover, a suspicious immune-mediated thickening plaque invaded the inferior temporal corneoscleral limbus and symblepharon immobilized the adjacent region (Fig. 1A). Trichiasis was noted over both upper and lower eyelid margin, and the upper conjunctival fornix foreshortening was significant. (Fig. 1B) Examination of oral mucosa and cutaneous revealed no erythematous, erosive lesions or blisters.

**Figure 2:**
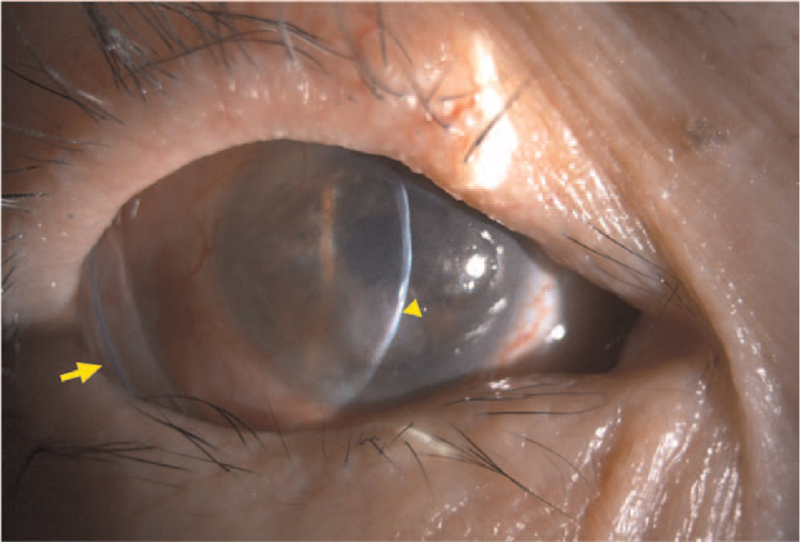
Slit lamp photograph of the right eye at postoperative two weeks. The perforated corneal sealed (arrowhead) and anterior chamber depth could be observed. Amniotic membrane started melting and ComfortRING^TM^ retained the reconstructed fornix spaces. (arrows).

Considering the patient's suspicious ocular history, the diagnosis of advance staging OCP with corneal perforation was impressed. Prophylactic antibiotic eye drops (0.5% Levofloxacin ophthalmic solution), artificial tears, and a bandage contact lens were applied. Ocular surface aerobic and anaerobic culture were obtained at initial presentation with no pathogen growth reported. Ocular surface reconstruction was smoothly done one week after initial encounter with the aid of sutureless cryopreserved amniotic membrane as the first layer and self-retained, cryopreserved amniotic membrane (PROKERA Slim) as the second. During the operation, the inflamed and fibrous tissue covering the cornea, bulbar conjunctiva, and the fornices were removed. The immune-mediated corneal plaque was partially excised, and pathology reported inflammatory changes. The corneal perforation site was sealed with tissue adhesive and fibrin glue, which was an alternative method to sutures, in order to secured the first layer amniotic membrane with the epithelium up and the basement membrane facing the ocular surface. The upper and lower fornix space were rebuilt with amniotic membrane and retained with the ComfortRING^TM^ of PROKERA Slim (Fig. 2).

**Figure 3:**
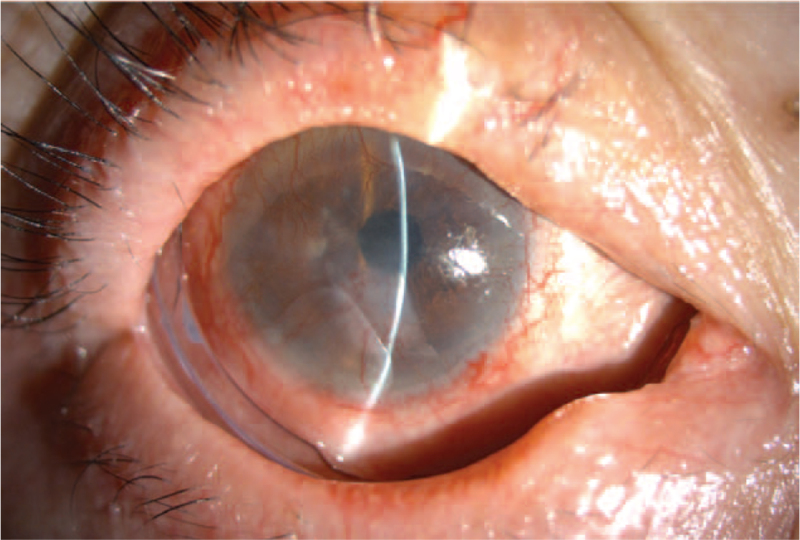
Slit lamp photograph of the right eye at postoperative one year follow up. Amniotic membrane has total melted. ComfortRING^TM^ was not removed for its function as a conformer.

Topical medications for postoperative care focused on inflammatory control, including ciclosporin (ikervis 1 mg/mL, two times/d), prednisolone acetate 1% (Pred-Forte 1% every 2 hours/d), and autologous serum eye drops (every hour/d). Systemic immunomodulatory therapy was not administered except for oral prednisolone (15 mg/d). The amnion membrane melted three weeks after the operation. Partial remission of the corneal neovascularization could be observed. No regrowth of symblepharon was noted. Topical medications were kept and reduced gradually. One months after the initial amniotic membrane transplantation (AMT) and reconstruction surgery, another two pieces of self-retained, cryopreserved amniotic membrane was applied consecutively under outpatient department setting. Slit-lamp examination revealed a stable ocular surface without sign of recurrence during the one year follow up (Fig. 3).

## 3 Discussion

OCP is a subset of systemic autoimmune disease mucous membrane pemphigoid, which extensively involve mucosal surfaces, such as the oral cavity, larynx, pharynx, esophagus, eye, nasal cavity, and genitalia.^[1,3]^ Classically, mucous membrane pemphigoid is characterized by a type II immune reaction depending on specific deposition of immunoreactants (IgG, IgA, IgM, and/or complement) along the epithelial basement membrane.^[1,3,5,6]^ This type II reaction leads to activation of the complement cascade provoking an intense inflammation. It is a potentially blinding rare systemic autoimmune disease with a reported incidence around 0.8 to 2 per million population and an average age at disease onset of 65 years.^[1,2,6]^

In OCP patients, the clinical manifestations are usually bilateral, asymmetrical and chronically progressive.^[4]^ The typical characteristics include corneal subepithelial blistering, conjunctival inflammation and eventually scarring. Secondary corneal neovascularization and fibrosis can also be found due to relapsing conjunctival inflammation. Eyelid abnormalities, such as trichiasis, entropion, and lagophthalmos, are often observed in OCP, which lead to the exposure and abrasion of the corneal surface and may confuse the diagnosis.

Early diagnosis is important. The revelation of linear deposition of IgA on epithelial basement membrane on a conjunctival biopsy with direct immunofluorescence is the gold standard in OCP diagnosis, though a negative pathology report does not exclude OCP.^[1,3,5,6]^ Therefore, OCP can still be diagnosed based on clinical characteristics even if the biopsy turns out to be negative. Since 1986, the Foster staging system classified OCP into four different stages clinically. Stage I denotes chronic conjunctivitis with subepithelial fibrosis and an unstable tear film. Stage II refers to inferior fornix foreshortening. While the Stage III describes symblepharon formation typically starting with the inferior fornix and the formation of ankyloblepharon with corneal scarring defines the end-stage, stage IV.^[5,7,8]^ An obvious symblepharon formation at the inferior fornix was seen in our patient which denotes stage III OCP.

The optimal therapeutic strategy is wide-ranging. In general, the main focus is to control the immune reaction and inflammation that leads to local ocular sequela of the disease. Currently, a “stepladder” approach is used to control OCP and its complications. Immunosuppressive agents including Dapsone, Methotrexate (MTX), Mycophenolate Mofetil (MMF), intravenous cyclophosphamide and Rituximab are crucial to suppress inflammation in patients with severe OCP.^[8]^ Among the systemic therapies used, Dapsone is preferred for mild to moderate OCP patients. But for patient who cannot tolerate Dapsone, MTX and MMF could be effective alternatives. Intravenous cyclophosphamide could be usable in severe OCP. For the aggressive forms of OCP or treatment failure groups, Rituximab should be considered.^[8,9]^ Despite the effectiveness of systemic immunosuppressant, discontinuation rates vary and poor compliance were still noted in some cases.^[8]^ In addition, OCP patients are general elderly with an advanced stage by the time of diagnosis. Using aggressive and long-term immunosuppressant may expose the patients to a higher risk of drug toxicity and side effects, including hematologic, gastrointestinal, cardiovascular and urinary complications.^[4,9]^

Besides, sole topical ciclosporin had been used to treat in small number of OCP patient with persistent inflammation despite conventional treatment of topical and/or systemic prednisolone. Although it seems to be sufficient to stop disease evolution, no conclusion can be drawn on the efficacy of topical anti-inflammatory treatment.^[9,10]^ In our patient, systemic immunosuppressive therapy was not used during the perioperative period due to the potential side effects and drug toxicity. Furthermore, we believed that topical ciclosporin and topical prednisolone aid in alleviating the conjunctival inflammation and shrinkage.^[10]^

For surgical interventions, ocular surgery should only be done in OCP patients after inflammation is completely under control in order to prevent incitement of relentless conjunctival inflammation and vicious cascade. In severe cases, autologous cultivated oral mucosal epithelial transplantation could be done but the intervention itself is technically demanding and unable to halt further cicatrization.^[11]^ Barabino S. et al. not only emphasized the supportive role of AMT in controlling inflammation and maintaining a stable conjunctiva, but also drew a conclusion in their prospective interventional noncomparative case series that AMT can be a first-step procedure for ocular surface reconstruction in OCP.^[12]^ Therefore, our treatment strategy mainly depends on the important characteristic of amniotic membrane.

In our patient, the first layer of amniotic membrane not only promoted the healing as a biological corneal bandage that serves as an ideal layer for normal epithelialization, but also suppressed the inflammation of the ocular surface. Another important consideration is that we applied the second layer of amniotic membrane, which prevents further mechanical abrasion related to trichiasis and lid-related complications. We chose the amniotic membrane product that comes with a ComfortRING^TM^ that acts as an ocular conformer which provides good support in maintaining a spacious reconstructed fornix. Systemic immunosuppressants were not prescribed in our patient as there were no signs of local recurrence during the one year follow-up period.

## 4 Conclusion

OCP is a rare and potentially blinding systemic autoimmune disease that affect mostly the elderly. Though in the recent decades, systemic immunosuppressants were reported to yield better outcomes in OCP treatment, side effects are unavoidable. Surgical interventions with double layer AMT demonstrated in conjunctival reconstruction in combination with topical anti-inflammatory medications seem to have a rational potential in treating OCP. In order to standardize the treatment protocol, more studies should be conducted.

## Author contributions

**Conceptualization:** Shu-Chun Kuo.

**Resources:** Shu-Chun Kuo

**Supervision:** Shu-Chun Kuo

**Writing – original draft:** Suan Hwang

**Writing – review and editing:** Suan Hwang, Shu-Chun Kuo
